# Compositional modeling of gas-condensate viscosity using ensemble approach

**DOI:** 10.1038/s41598-023-36122-3

**Published:** 2023-06-14

**Authors:** Farzaneh Rezaei, Mohammad Akbari, Yousef Rafiei, Abdolhossein Hemmati-Sarapardeh

**Affiliations:** 1 Department of Petroleum Engineering, Amirkabir University of Technology, Tehran, Iran; 2 Department of Mathematics and Computer Science, Amirkabir University of Technology (Tehran Polytechnic), Tehran, Iran; 3 Department of Petroleum Engineering, Shahid Bahonar University of Kerman, Kerman, Iran; 4 State Key Laboratory of Petroleum Resources and Prospecting, China University of Petroleum (Beijing), Beijing, China

**Keywords:** Energy science and technology, Engineering

## Abstract

In gas-condensate reservoirs, liquid dropout occurs by reducing the pressure below the dew point pressure in the area near the wellbore. Estimation of production rate in these reservoirs is important. This goal is possible if the amount of viscosity of the liquids released below the dew point is available. In this study, the most comprehensive database related to the viscosity of gas condensate, including 1370 laboratory data was used. Several intelligent techniques, including Ensemble methods, support vector regression (SVR), K-nearest neighbors (KNN), Radial basis function (RBF), and Multilayer Perceptron (MLP) optimized by Bayesian Regularization and Levenberg–Marquardt were applied for modeling. In models presented in the literature, one of the input parameters for the development of the models is solution gas oil ratio (Rs). Measuring Rs in wellhead requires special equipment and is somewhat difficult. Also, measuring this parameter in the laboratory requires spending time and money. According to the mentioned cases, in this research, unlike the research done in the literature, Rs parameter was not used to develop the models. The input parameters for the development of the models presented in this research were temperature, pressure and condensate composition. The data used includes a wide range of temperature and pressure, and the models presented in this research are the most accurate models to date for predicting the condensate viscosity. Using the mentioned intelligent approaches, precise compositional models were presented to predict the viscosity of gas/condensate at different temperatures and pressures for different gas components. Ensemble method with an average absolute percent relative error (AAPRE) of 4.83% was obtained as the most accurate model. Moreover, the AAPRE values for SVR, KNN, MLP-BR, MLP-LM, and RBF models developed in this study are 4.95%, 5.45%, 6.56%, 7.89%, and 10.9%, respectively. Then, the effect of input parameters on the viscosity of the condensate was determined by the relevancy factor using the results of the Ensemble methods. The most negative and positive effects of parameters on the gas condensate viscosity were related to the reservoir temperature and the mole fraction of C_11_, respectively. Finally, suspicious laboratory data were determined and reported using the leverage technique.

## Introduction

The process of hydrocarbon production is associated with a continuous reduction of reservoir pressure. By reducing the reservoir pressure below the dew point pressure, the condensate gas reservoir composition changes from a single-phase gas to a two-phase gas–liquid state. The liquid phase produced is the valuable condensate that basically cannot move and produce spontaneously^1^. With continuous production from the gas condensate reservoir and further reduction of the reservoir pressure, condensate accumulates in the area around the wellhead and causes the production wellhead to be blocked and the gas production rate to be drastically reduced. On the other hand, these condensate compounds, which are known as rich compounds, remain in the reservoir. Condensate saturation is a function of fluid properties that affect the rate of reservoir production. One of the most important properties of fluid is viscosity. Providing an accurate model that well describes the phase behavior of the reservoir has a special place in economic projects and reservoir production plans^2^. The multiphase flow in condensate gas reservoirs is due to reduction of the pressure below the dew point pressure and conversion of some heavy gases into liquid. In condensate-rich gas reservoirs, the accumulation of these liquids in the area around the well gradually increases, which reduces the performance of the reservoir. In order to solve this problem in these reservoirs, an attempt is made to prevent the reservoir pressure from falling below the dew point pressure or to produce gas condensate created in the area around the well; thus, it is important to accurately predict viscosity. In fact, inaccurate estimation of condensate liquid viscosity below the dew point has a detrimental effect on cumulative production and can lead to large errors in reservoir performance. Previous studies show 1% error in reservoir fluid viscosity resulted in 1% error in cumulative production^3–5^.

Viscosity is a measure of the internal friction or flow resistance of fluid and occurs when there is relative motion between fluid layers. Viscosity is caused by the following two factors^6^:

A) Molecular gravitational forces that occur in liquids.
B) Momentum exchange forces of molecules in gases.

Viscosity is the measure of fluid resistance to flow. The general unit of metric for absolute viscosity is Poise, which is defined as the force required to move one square centimeter from one surface to another in parallel at a speed of one centimeter per second (cm/s). A film is separated from the fluid with a thickness of one centimeter. For ease of use, centipoise (cp) (one-hundredth of a pup) is the usual unit used. In the laboratory, gravity is typically used to measure viscosity to create flow through a temperature-controlled capillary tube (viscometer). This measurement is called kinematic viscosity. The unit of kinematic viscosity is the stoke, which is expressed in square centimeters per second. The more commonly called unit is the cent stake (CST)^7^.

To date, efforts have been made to predict the viscosity of gas condensate under different conditions. Lohrenz et al.^8^ predicted the viscosity of gas condensate based on the fluid composition used^8^. Lohrenz model has been used in industry due to its high accuracy in predicting viscosity and is known as LBC. This model was first used to predict the viscosity of heavy gas mixture^9^. The LBC model is accurate for predicting gas viscosity in condensate/gas reservoirs but is not accurate enough to predict liquid phase viscosity, and therefore changing the coefficients of this model is necessary to increase accuracy^3^. Yang et al.^3^ proposed a model for predicting fluid viscosity that is a function of reservoir pressure and temperature, gas/oil ratio (GOR), and specific gravity of the gas. Then Dean and Steele’s^10^ model was presented for gas mixtures. The main application of this model is in moderate and high-pressure conditions. The model was developed using the critical constants and molecular weight of the components and is a function of temperature and pseudo-reduced pressure. Hajirezaei et al.^11^ also presented an accurate model for calculating the viscosity of gas mixtures using gene expression programming (GEP) based on reduced temperature and pressure. Furthermore, different mathematical models have been proposed to predict the viscosity of gas mixture in different ranges of temperature, pressure, specific gravity, GOR, and liquid viscosity^12–15^. All of these models, which estimate the viscosity in the liquid phase, are used for oil and are a function of the viscosity of the crude oil, which is very different from the liquid of the condensate reservoirs and is not suitable for predicting the viscosity of condensate^16^. Also, due to the variability of viscosity in condensate reservoirs due to pressure changes, the empirical relationships provided to estimate the viscosity of gas mixture cannot well describe the behavior of condensate^4,17,18^.

In recent years, the use of machine learning methods has been widely increased in the oil industry due to its ability to solve complex problems and very high accuracy. To date, these methods have been used to estimate GOR, dew point pressure, and other characteristics of condensate gas reservoirs, the main of which is to predict dew point pressure^19–22^. As examples, in a research by Onwuchekwa^23^, the application of machine learning was discussed to estimate the properties of reservoir fluids. The models used in it include K-nearest neighbors (KNN), support vector machine (SVM) and random forest (RF), and 296 data were used to estimate reservoir fluid properties. Also, to predict the relative permeability of condensate gas reservoirs, an accurate model was recently presented by Mahdaviara et al.^24^ using machine learning methods. After that, Mouszadeh et al.^25^ estimated the viscosity of condensate using Adaptive Neuro Fuzzy Inference System-Particle Swarm Optimization (ANFIS-PSO) and Extreme Learning Machine (ELM) and concluded that the ELM model is more accurate. Finally, Mohammadi et al.^26^ investigated the effect of velocity on relative permeability in condensate reservoirs in the absence of inertial effects. Since the prediction of gas viscosity in gas condensate reservoirs has great importance and its accurate measurement has a special effect on cumulative production, in this paper, we have tried to model the viscosity of gas condensate using different algorithms and a complete database.

As mentioned, estimating the viscosity of gas condensate is a critical issue in the oil industry because by using this parameter, the flowrate of reservoirs can be estimated. Therefore, the accurate estimation of this parameter leads to the accurate estimation of the flowrate of gas reservoirs and checking their performance. For this reason, in this research, using a wide database including 1370 laboratory data, accurate compositional models are presented to estimate this parameter. The dataset is divided into two categories of training and testing in the form of 80/20. Temperature, pressure, and condensate compositions are used as inputs to the models. In the literature, the input data for the development of the models included temperature, pressure, solution gas oil ratio (Rs) and reservoir fluid composition. Also, some of the models presented in the literature were not highly accurate, and in some researches, a limited database was used. In this research, in addition to using a large database, some models with high accuracy were presented. Intelligent models including Ensemble methods, Support vector regression (SVR), K-nearest neighbors (KNN), Radial basis function (RBF), and Multilayer Perceptron (MLP) optimized by Bayesian Regularization (BR) and Levenberg–Marquardt (LM) are used for modeling the gas-condensate viscosity. Using the error parameters and graphical diagrams, the presented models are evaluated and finally, the effect of input parameters on the most accurate model is investigated and suspicious laboratory data are identified using the leveraging technique.

## Data gathering

In this study, a comprehensive set of data was collected to predict the viscosity of gas condensate^4,27–36^. The data set includes 1370 laboratory data points comprising of temperature and pressure of gas reservoirs and components of condensate mixtures (from C_1_ to C_11_ and the molecular weight of C_12+_ along with N_2_ and CO_2_), which are the inputs of the models. The statistical parameters of the data used are shown in Table 1.

**Table 1:** Statistical Parameters of the used dataset.

	Average	Min	Max	Median	Mode	Kurtosis	Skewness	Standard deviation
Temperature, K	371.396	151.000	639.000	378.000	403.150	0.458	0.065	74.085
Pressure, MPa	33.102	0.020	138.060	30.150	0.101	3.763	1.465	23.752
N_2_, mole %	0.004	0.000	0.071	0.000	0.000	25.893	4.509	0.009
CO_2_, mole %	0.016	0.000	0.079	0.000	0.000	0.467	1.519	0.030
C_1_, mole %	0.415	0.000	0.898	0.410	0.100	−1.778	0.020	0.318
C_2_, mole %	0.013	0.000	0.131	0.000	0.000	7.844	2.663	0.026
C_3_, mole %	0.292	0.000	0.900	0.011	0.000	−1.381	0.785	0.415
C_4_, mole %	0.009	0.000	0.065	0.000	0.000	4.981	2.384	0.017
C_5_, mole %	0.012	0.000	0.133	0.000	0.000	9.505	3.315	0.033
C_6_, mole %	0.007	0.000	0.059	0.000	0.000	5.294	2.550	0.016
C_7_, mole %	0.035	0.000	0.580	0.000	0.000	21.250	4.405	0.094
C_8_, mole %	0.003	0.000	0.030	0.000	0.000	7.162	2.948	0.008
C_9_, mole %	0.003	0.000	0.032	0.000	0.000	8.665	3.206	0.008
C_10_, mole %	0.136	0.000	1.000	0.013	0.000	3.558	2.053	0.244
C_11_, mole %	0.003	0.000	0.040	0.000	0.000	10.189	3.463	0.010
Molecular Weight of C_12+_	65.654	0.000	271.000	0.000	0.000	−1.443	0.707	91.787

## Model development

### Support vector regression (SVR)

The use of support vector machines (SVM) provided by Vapnik^37^ has been developed as a solution to machine learning and pattern recognition. SVM makes its predictions using a linear combination of the Kernel function that acts on a set of training data called support vectors. The characteristics of an SVM are largely related to its kernel selection.

**Figure 1:**
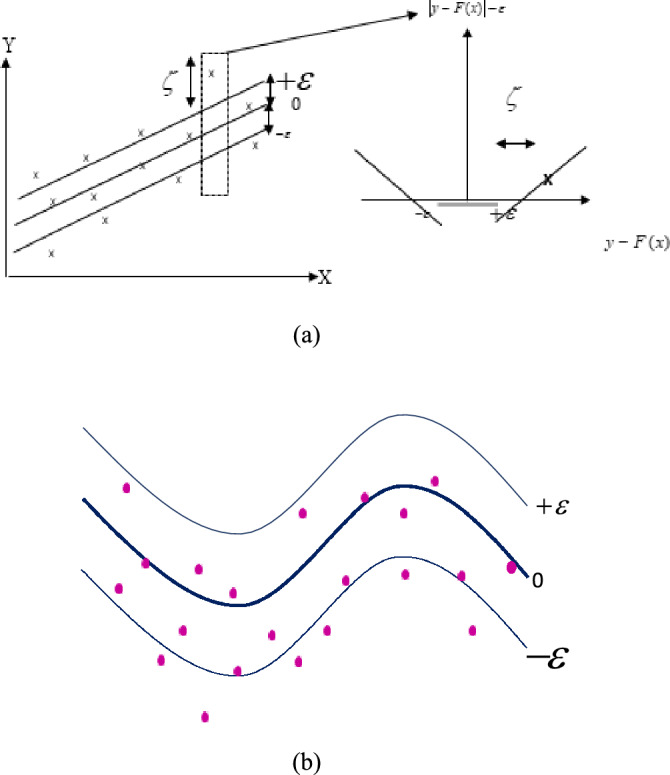
Schematic of the proposed SVR; (**a**) linear and (**b**) nonlinear function.

By defining a ε-sensitive region across the function, SVM is generalized to SVR. Moreover, this ε solves the optimal problem again and estimates the target value in such a way that the model complexity and the model accuracy value are balanced. The SVR algorithm is one of the machine learning algorithms; which is based on the theory of statistical education. This method, which is one of the supervised training methods, establishes a relationship between the input data and the value of the dependent parameter, based on structural risk minimization^38^. Classical statistical methods are superior and, unlike methods such as neural networks, do not converge to local responses. SVR is a method for estimating a function that is mapped to a real number based on training data from an input object. A multidimensional space is mapped; then a super plane is created that separates the input vectors as far apart as possible^39^. A kernel function is used to solve the problem of operating in a large space, in which case the operation can be performed. Input the data space with the same speed as the kernel function, in fact, the problem of multidimensional and nonlinear mapping is solved^40^. The optimization process must be accompanied by a modified drop function to include the distance measurement. In fact, the purpose of the SVR is to estimate the parameters of weights and bias is a function that best fits the data^41^. The SVR function can be linear (Fig. 1a) or nonlinear (Fig. 1b) and the nonlinear model is the calculation of a regression function in a high-dimensional feature space in which input data is represented by a nonlinear function.

Assuming that there is training data if each input *X* has several *D* attributes (in other words, belongs to a space with dimension *D*) and each point has a value of *Y*—like all regression methods—the goal of finding a function is to establish a relationship between input and output^42^.

1 $$f(x,w) = w^{T} x + b$$

To obtain the function *f*, it is necessary to calculate the values of *w* and *b*. To calculate the values of *w* and *b*, the next relationship must be minimized^37^.

2 $$R(C) = \frac{1}{2}\left\| w \right\|^{2} + C\frac{1}{l}\sum\limits_{i = 1}^{l} {L_{\varepsilon } (y_{i} ,f_{i} (x,w))}$$

where *C* is a constant parameter and its value must be specified by the user. In fact, the function of the constant *C* parameter is to create equilibrium and change the weights of the amount of the fine due to negligence (variable $\varepsilon$) and at the same time to maximize the size of the separation margin. The Lc function is the Vpnik function, which is defined as follows^43^:

3 $$\left| {y - f(x,w)} \right|_{\varepsilon } = \left\{ {\begin{array}{*{20}l} 0 \hfill & {{\text{if}}\,\,\left| {y - f(x,w)} \right| \le \varepsilon } \hfill \\ {\left| {y - f(x,w)} \right| - \varepsilon } \hfill & {{\text{Otherwise}}} \hfill \\ \end{array} } \right.$$

The above problem is rewritten to maximize the following equation:

4 $$L_{p} (a_{i} ,a_{i}^{*} ) = - \frac{1}{2}\sum\limits_{i,j = 1}^{l} {(a_{i} - a_{i}^{*} )(a_{j} - a_{j}^{*} )x_{i}^{T} x_{j} - \varepsilon \sum\limits_{i = 1}^{l} {(a_{i} + a_{i}^{*} ) + } } \sum\limits_{i = 1}^{l} {(a_{i} - a_{i}^{*} )}$$

The conditions are as follows:

5 $$\left\{ \begin{gathered} \sum\limits_{i = 1}^{l} {(a_{i} - a_{i}^{*} )} = 0 \hfill \\ 0 \le a_{i} \le C,\quad i = 1,...,l \hfill \\ 0 \le a_{i}^{*} \le C,\quad i = 1,...,l \hfill \\ \end{gathered} \right.$$

By solving the above equation, the SVR function, i.e., *f*, can be calculated using the kernel function as follows:

6 $$f(x,w) = w_{0}^{T} x + b = \sum\limits_{i = 1}^{l} {(a_{i} - a_{i}^{*} )x_{i}^{T} } x + b$$

Support Vector Machines (SVM) is a widely used supervised learning algorithm in the field of machine learning, which is based on the principle of maximizing the margin between the different classes^44^. The assumptions and limitations of SVM are as follows:

Assumptions:

Large Margin: SVM assumes that it is better to consider a large margin while separating the classes to achieve better generalization performance^44^.

Support Vectors: SVM relies on support vectors, which are crucial data points that determine the boundary between the classes. Accurate selection of these points is important to achieve good modeling results^44^.

Limitations:

Large Datasets: SVM is not well-suited for very large datasets as the time required to train the model increases significantly with the size of the dataset^45^.

High Noise: SVM can be sensitive to high levels of noise in the dataset, which can affect the accuracy of the model, particularly in the case of Support Vector Regression (SVR)^45^.

In summary, while SVM has certain assumptions and limitations, it remains a popular and effective machine learning algorithm for a wide range of applications. However, it is important to carefully consider the limitations and suitability of SVM for specific datasets and problems^45^.

### K-nearest neighbors (K-NN)

KNN regression is a nonparametric regression that was first used by Karlsson and Yakowitz in 1987 ^46^ to predict and estimate hydrological variables. In this method, a predetermined parametric relationship is not established between the input and output variables, but in this method, to model a process, the information obtained from the observational data is used based on the similarity between the desired real-time variables and the observational period variables^38^. The logic used in this method is to calculate the probability of an event occurring based on similar historical events (observational events). In this method, to determine the similarity of current conditions to historical conditions, the kernel *f*(*D*_*ri*_) probability function is used as follows^47^:

7 $$f(D_{ri} ) = \frac{{{\raise0.7ex\hbox{$1$} \!\mathord{\left/ {\vphantom {1 {D_{ri} }}}\right.\kern-0pt} \!\lower0.7ex\hbox{${D_{ri} }$}}}}{{{\raise0.7ex\hbox{${\sum\limits_{i = 1}^{k} 1 }$} \!\mathord{\left/ {\vphantom {{\sum\limits_{i = 1}^{k} 1 } {D_{ri} }}}\right.\kern-0pt} \!\lower0.7ex\hbox{${D_{ri} }$}}}}$$

where *D*_*ri*_ is the Euclidean distance of the current condition vector (*X*_*r*_) from the historical observational vector (*X*_*i*_) and K is the number of neighborhoods closest to the current condition. The output of this regression model (*Y*_*r*_) for the input vector *X*_*r*_ is calculated based on the above kernel relation and the corresponding *Y*_*i*_ values for each *D*_*ri*_ from the following relation^47^:

8 $$Y_{r} = \sum\limits_{i = 1}^{k} {f(D_{ri} ) \times Y_{i} }$$

**Figure 2:**
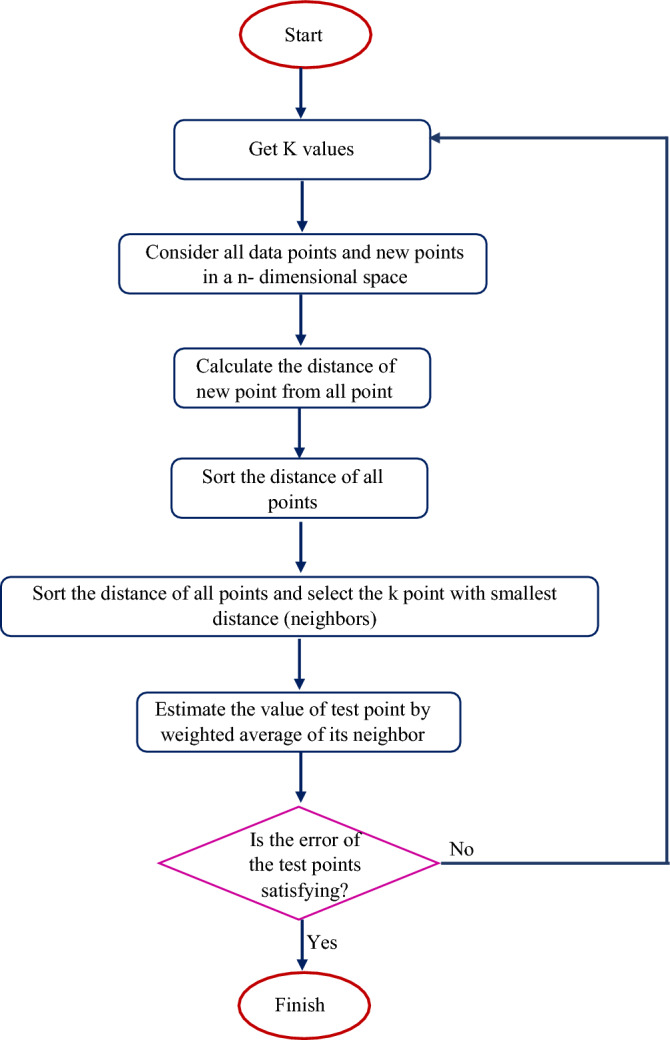
Flowchart of K-NN algorithm used in this study.

In the KNN model, the choice of the number of nearest neighbors (K) affects the accuracy of the results, so that if the number of neighbors is large, the results are close to the average of the observational data, and if it is very small, the possibility of increasing the error increases^48^. Therefore, determining the optimal number of this parameter in this model is necessary to achieve the least error. Figure 2 shows the flowchart of the KNN algorithm used in this research.

The advantages of using the KNN algorithm in prediction processes can be mentioned as follows^49^:

1. Simple execution.
2. No need to estimate the parameters.
3. Non-linear modeling capability.
4. Effectiveness and performance with high efficiency in the face of a large number of data sets.

Limitations of using the K-NN algorithm in predictive processes include the following:

Since this model tries to identify similar patterns in time series and use them in forecasting, sufficient information is necessary to validate it. Short-term information can lead to many errors in modeling using this algorithm. As can be seen from the relationships related to the structure of the K-NN method for estimating information by this algorithm, this algorithm is not capable of producing values greater than the most historically observed value and less than the least observed observational value. In other words, this algorithm only has the ability to interpolate information and is not capable of extrapolation. Therefore, the use of this algorithm in predicting values may to some extent lead to significant errors^50^.

The K-Nearest Neighbors (KNN) algorithm has certain assumptions and limitations that should be taken into consideration.

Assumptions:

Local Similarity: This assumption is important since the algorithm determines the class of a data point based on the classes of its nearest neighbors. Full explanation regarding this assumption are mentioned above^47^.

Relevant Features: The algorithm assumes that all features used in the model are equally relevant and contribute to the prediction task. This may not always be the case in real-world scenarios, as some features may have more impact on the target variable than others^47^.

Limitations:

Parameter Tuning: One limitation of the KNN algorithm is the need to determine the value of K, which can be a complex process. Choosing the wrong value for K can lead to overfitting or underfitting of the model, resulting in poor performance^38^.

High Computational Cost: The algorithm requires computing the distances between the query point and all the data points, which can be computationally expensive, particularly with large datasets. The high computational cost can limit the scalability of the algorithm for large datasets^38^.

In summary, the KNN algorithm has assumptions and limitations that need to be considered while using it. It is essential to choose the appropriate value for K and consider the computational cost when using the algorithm on large datasets^38^.

### Ensemble learning

**Figure 3:**
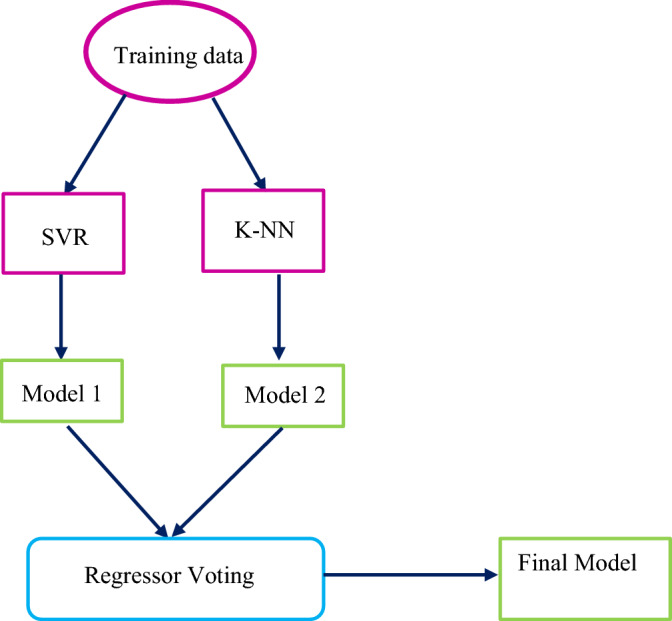
Schematic of the proposed Ensemble methods.

In machine learning, the combined methods of algorithms are used to better predict the results than the individual results of each algorithm. The models used in this set are limited and specific but form a flexible structure and this algorithm reports better results when there is a lot of variation between the models used. Variation in the training phase for regression is done by correlation and for classification using cross-entropy^51–53^. Figure 3 shows the ensemble flowchart method used in this research. The following item is the most widely used ensemble method.

#### Bayesian model averaging

In the Bayesian model averaging method, known as BMA, predictions are made by averaging the weights given to each model. The BMA method is more accurate than single models when different models perform the same function during training^54^. The greatest understandable question with any method that usages Bayes' theorem is the prior, i.e., a specification of the likelihood (subjective, perhaps) whether every model is the most accurate or not. Theoretically, BMA is utilized by each prior. In Bayesian probabilistic space, for hypothesis h, the conditional probability distribution is defined as^55^:

9 $${\text{h}}({\text{x}}) = {\text{P}}\left( {{\text{f}}({\text{x}}) = {\text{y}}|{\text{x}},{\text{h}}} \right)$$

Using the point *x* and the training sample *S*, the forecast of the function *f*(*x*) can be calculated oppositely:

10 $${\text{P}}({\text{f}}({\text{x}}) = {\text{y}}|{\text{S}},{\text{x)}}$$

It can also be rewritten as a weighted sum of all hypotheses. This problem can be considered as an ensemble problem consisting of hypotheses in *H*, each of which is weighted by its posterior probability *P* (*h | S*). In Bayesian law, the posterior probability is proportional to the likelihood multiplication of the training data in the prior probability *h: P* (*h | S*) ∝ *P* (*S | h*)* P* (*h*).

Also, in some cases, the Bayesian committee can be calculated by considering and calculating *P* (*S | h*) and *P* (*h*). Also, if the correct function *f* is selected from *H* according to *P* (*h*) then Bayesian voting works optimally^54^.

Bayesian Model Averaging (BMA) is an ensemble modeling technique that includes certain assumptions and limitations that should be taken into account.

Assumptions:

Model Independence: BMA assumes that the models in the ensemble are independent of each other, and their errors are uncorrelated^54^.

Model Fit: The ensemble model assumes that each model is well-suited to the dataset and provides accurate predictions^54^.

Limitations:

Hyperparameter Selection: One of the main limitations of the ensemble model is the challenge of selecting the hyperparameters for each individual model. The wrong choice of hyperparameters can lead to lower accuracy than the individual models^54^.

Time and Space Complexity: BMA requires more computational resources and time than individual models, as it uses multiple algorithms simultaneously. This can be a limitation when working with large datasets or limited computational resources^55^.

In summary, Bayesian Model Averaging is an effective technique for ensemble modeling, but it has certain assumptions and limitations that should be considered. Proper selection of hyperparameters and computational resources are important factors for achieving good performance with the ensemble model^55^.

### Multi-layer perceptron (MLP)

One of the most common types of neural networks is the multilayer perceptron (MLP). This network consists of an input layer, one or more hidden layers, and an output. MLP can be trained by a backward propagation algorithm^56^. Typically, MLP is organized as an interconnected layer of input, hidden, and output artificial neurons. Then, by comparing network output and actual output, the error value is calculated, and this error is returned as BP in the network to reset the connecting weights of the nodes. The BP algorithm consists of two steps; in the first step the effect of network inputs is pushed forward to reach the output layer. The error value is then reversed and distributed in the network^57^.

In each layer, a number of neurons are considered that are connected to the neurons of the adjacent layer by connections. It should be noted that the number of intermediate layers and the number of neurons in each layer should be determined by trial and error by the designer^6^.

The error in the output node *j* is shown as the nth point of the data. Where *d* is the target value and *y* is the value produced by perceptron.

11 $$MSE = \frac{{\sum_{j = 0}^{P} {\sum_{i = 0}^{N} {\left( {d_{ij} - y_{ij} } \right)^{2} } } }}{N}$$

Node values are adjusted based on corrections that minimize the total error rate as follows^58^:

12 $$\varepsilon (n) = \frac{1}{2}\sum\nolimits_{j} {e_{j}^{2} (n)}$$

Using the gradient, the change in weight is as follows:

13 $$\Delta \omega_{ji} (n) = - \eta \frac{\partial \varepsilon (n)}{{\partial v_{j} (n)}}y_{i} (n)$$

where *y*_*i*_ is the output of the former neuron and the amount of learning that is chosen to ensure that the weights converge rapidly to the more accurate response. The calculated derivative depends on the induced local field *v*_*j*_, which itself changes. It is easy to prove that this derivative can be simplified for the output node.

14 $$- \frac{\partial \varepsilon (n)}{{\partial v_{j} (n)}} = e_{j} (n)\varphi^{^{\prime}} (v_{j} (n))$$

where $\varphi^{^{\prime}}$ is a derivative of the activation function and does not change itself. The analysis is more difficult to change the weights to a hidden node, but the corresponding derivative can be shown as follows:

15 $$- \frac{\partial \varepsilon (n)}{{\partial v_{j} (n)}} = \varphi^{^{\prime}} (v_{j} (n))\sum_{k} {\frac{\partial \varepsilon (n)}{{\partial v_{k} (n)}}} \omega_{kj} (n)$$

**Figure 4:**
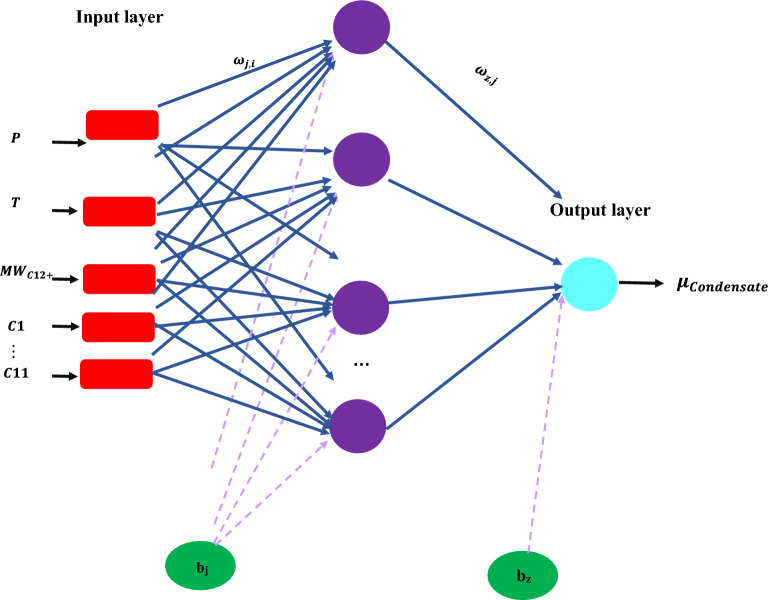
MLP structure proposed in this research.

This depends on the change in weight of the nodes that represent the output layer; therefore, to change the hidden layer weights, the output layer changes according to the derivative of the activation function, and thus this algorithm shows a function of the activation function^59^. Figure 4 shows the MLP structure presented in this research.

Multilayer Perceptron (MLP) is a widely used artificial neural network model to extract and learn features from the data. However, there are certain assumptions and limitations that should be considered when using MLP^56^.

Assumptions:

Dense Connectivity: The MLP model assumes that neurons in consecutive layers are densely connected, meaning that all input values are passed to the next neuron, and their output is then sent to the neurons in the next layers^56^.

Limitations:

Large Number of Parameters: MLP can have a large number of parameters, particularly when using multiple hidden layers or large input sizes, resulting in increased model complexity and longer training times. This can be a limitation when working with limited computational resources or large datasets^57^.

Overfitting: Due to the large number of parameters, MLP is prone to overfitting, particularly when working with small datasets or complex models. Regularization techniques such as dropout or weight decay can be used to mitigate this limitation^57^.

In summary, MLP is a powerful machine learning model with certain assumptions and limitations. Dense connectivity between neurons and the large number of parameters used are important factors to consider when using MLP. Careful selection of the model architecture and regularization techniques can help to achieve better performance and prevent overfitting^6^.

#### Bayesian Regularization (BR) Algorithm

BR algorithm is a backpropagation error method. The backpropagation network training process with the BR algorithm begins with the random distribution of initial weights. Distribution Randomization of these parameters determines the initial orientation before providing data to the network. After giving data to the network, optimization of primary weights is started until a secondary distribution is obtained using BR since the data used may be associated with many errors, effective methods will be necessary to improve the generalization performance. Hence, the BR includes network complexity regulation and modifying performance function^60,61^.

#### Levenberg–Marquardt (LM) Algorithm

This algorithm, also called TRAINLM, is one of the fastest back-propagation algorithms that uses standard numerical optimization techniques. This method tries to reduce the calculations by not calculating the Hessian matrix of the second derivative of the data matrix. When the performance function is the sum of the squares common in leading networks, the Hessian matrix can be estimated using the following Eqs. ^62^. In this relation, *J* is the Jacobin matrix, which contains the first derivatives of network errors relative to weights and biases, and *e* is the network error vector. The Jacobin matrix can be calculated using standard back-propagation techniques, and its computational complexity is much less than that of the Hessian matrix^63^.

16 $$H = JJ^{T}$$

17 $$g = eJ^{T}$$

Like other numerical algorithms, the LM algorithm has an iterative cycle. In a way that starts from a starting point as a conjecture for the vector *P* and in each step of the iterative cycle the vector *P* is replaced by a new estimate *q* + *p* in which the vector *q* is obtained from the following approximation^63^:

18 $$f(p + q) \approx f(p) + Jq$$

In the above equation, *J* is Jacobin *f* in *P* that there is a network weights optimizing process in the problem of the sum of squares *S*: $\nabla_{q} S = 0$.

By linearizing the above formula, the following equation can be obtained:

19 $$(J^{T} J)q = - J^{T} f$$

In the above formula, q can be obtained by inverting ($J^{T} J$)^64^.

### Radial Basis Function (RBF)

The RBF neural network has a very strong mathematical basis based on the hypothesis of regularity and is known as a statistical neural network. In general, this network consists of three layers including input, hidden, and output. In the hidden layer, the Gaussian transfer function is used and in the output layer, it is a linear transfer function. In fact, the neuron of the RBF method is a Gaussian function. The input of this function is the Euclidean distance between each input to the neuron with a specified vector equal to the input vector^65^. Equation (19) shows the general form of the output neurons in the RBF network^65^.

20 $$C_{j} (x) = \sum\limits_{i = 1}^{k} {w_{ji} \phi \left( {\left\| {x - \mu_{i} } \right\|;\sigma_{i} } \right)}$$

where in this equation:

$C_{j} (x)$: function dependent on j^th^ output,

K: number of radial basis function,

$\phi$: radial basis function with $\mu_{i}$ center and $\sigma_{i}$ bandwidth,$w_{ji}$: the weight depends on the j^th^ class and the i^th^ center,

$\phi \left( {\left\| {x - \mu_{i} } \right\|;\sigma_{i} } \right)$: radial basis function and || || means Euclidean distance.

**Figure 5:**
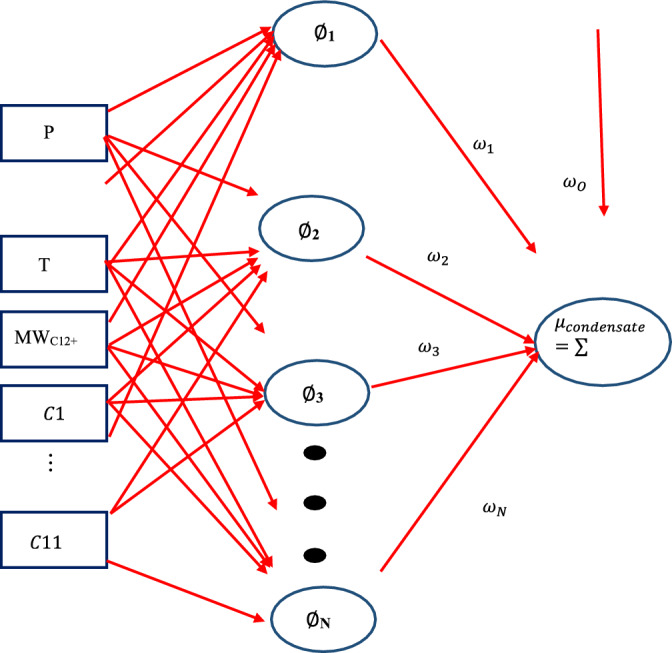
RBF structure utilized to predict gas-condensate viscosity.

In the RBF network, the distance between each pattern and the center vector of each neuron in the middle layer is calculated as a radial activation function^66,67^. The RBF flowchart used in this research is presented in Fig. 5.

Radial Basis Function (RBF) is a widely used machine learning algorithm. However, there are certain assumptions that should be taken into consideration when using RBF.

Assumptions:

Two-Layer Neural Network: RBF assumes a two-layer neural network architecture, consisting of a hidden layer with radial activation functions and an output layer that computes the weighted sum of the hidden layer's outputs^65^.

Radial Activation Functions: RBF uses radial activation functions in the hidden layer, which are centered on specific points in the input space and have a bell-shaped activation function^65^.

Nonlinear Inputs, Linear Outputs: RBF assumes that the inputs are nonlinear and that the outputs are linear, meaning that the model can capture nonlinear relationships between the input features, while still providing a linear output^65^.

Limitations:

Scalability: RBF can be computationally expensive and challenging to scale for large datasets or high-dimensional feature spaces^60^.

Sensitivity to Hyperparameters: RBF requires careful selection of hyperparameters, such as the number of radial basis functions and their centers, which can impact the model's performance^60^.

In summary, RBF is a powerful algorithm that assumes a two-layer neural network with radial activation functions in the hidden layer and linear outputs. However, it has certain limitations such as scalability and sensitivity to hyperparameters. Proper selection of hyperparameters and careful consideration of the computational resources required are important factors to consider when using RBF^60^.

## Results and discussion

In this study, using different algorithms including Ensemble-Methods, SVR, KNN, RBF, and MLP neural network trained with BR and LM algorithms, several models were presented for predicting the viscosity of gas condensate. The time required for running and the hyper-parameters related to each model are reported in Table 2. The statistical parameters of error used in this study to check the accuracy of the models include standard deviation (SD), average percent relative error (APRE, %), determination coefficient (R^2^), average absolute percent relative error (AAPRE, %), and root mean square error (RMSE) as defined below^68^:

21 $$APRE=\frac{100}{N}\sum_{i=1}^{N}\left(\frac{{\mu }_{{g}_{i}}^{act}-{\mu }_{{g}_{i}}^{cal}}{{\mu }_{{g}_{i}}^{act}}\right)$$

22 $$RMSE={\left(\frac{\sum_{i=1}^{N}{\left({\mu }_{{g}_{i}}^{act}-{\mu }_{{g}_{i}}^{cal}\right)}^{2}}{N}\right)}^\frac{1}{2}$$

23 $$AAPRE=\frac{100}{N}\sum_{i=1}^{N}\left|\frac{{\mu }_{{g}_{i}}^{act}-{\mu }_{{g}_{i}}^{cal}}{{\mu }_{{g}_{i}}^{act}}\right|$$

24 $$SD={\left(\frac{1}{N-1}\sum_{i=1}^{N}{\left(\frac{{\mu }_{{g}_{i}}^{act}-{\mu }_{{g}_{i}}^{cal}}{{\mu }_{{g}_{i}}^{act}}\right)}^{2}\right)}^\frac{1}{2}$$

25 $$R - squared(R^{2} ) = 1 - \frac{{\sum_{i = 1}^{N} {(\mu_{i}^{act} - \mu_{i}^{cal} )^{2} } }}{{\sum_{i = 1}^{N} {(\mu_{i}^{act} - \overline{{\mu^{act} }} )^{2} } }}$$

**Table 2:** Hyper-parameters and run time of developed models.

Model	Hyper-parameters	Run time (min)
Ensemble methods	SVR + K-NN C = 200, $\varepsilon = 0.00001$ K-neighbours = 2	3
SVR	C = 200, $\varepsilon = 0.00001$	5
K-NN	K-neighbours = 2	2
MLP-LM	Transfer function = Tansig-Tansig Number of neurons = 10,12	80
MLP-BR	Transfer function = Tansig-Tansig Number of neurons = 10,12	55
RBF	Max neuron = 300 Spread = 1.5	15

### Precisions and validities of the models

Table 3 is presented to evaluate the accuracy of the models developed in this study using statistical error parameters calculated for training, test, and total data. According to the results presented in this table, it can be concluded that Ensemble methods showed a small AAPRE and the difference between train error and test error in this model is less than in the other developed models. The calculated AAPRE for this algorithm is 4.83% and its other error parameters are as follows: R^2^ = 0.9781, APRE = −0.05%, SD = 0.031966, and RMSE = 0.044646.

**Table 3:** Statistical parameters of the proposed models for determination of viscosity of gas condensate.

Model		APRE, %	AAPRE, %	RMSE	SD, %	R^2^
Ensemble methods	Train	−0.712	4.58	0.043951	0.031448	0.9805
	Test	−0.068	5.86	0.047429	0.034041	0.9695
	Total	−0.55	4.83	0.044646	0.031966	0.9788
SVR	Train	0.45	4.56	0.053017	0.024128	0.9719
	Test	0.40	6.49	0.047857	0.044276	0.9692
	Total	0.44	4.95	0.051985	0.0281576	0.9715
K-NN	Train	−1.93	5.24	0.044429	0.089516	0.9800
	Test	−0.23	6.31	0.053367	0.060065	0.9612
	Total	−1.59	5.45	0.046217	0.083579	0.9771
MLP-LM	Train	−2.96	8.11	0.032649	0.026572	0.9883
	Test	−1.80	7.10	0.055282	0.017382	0.971
	Total	−2.71	7.89	0.038174	0.024672	0.9844
MLP-BR	Train	−2.34	6.42	0.020271	0.021717	0.9956
	Test	−0.03	7.35	0.055470	0.038123	0.9679
	Total	−1.92	6.56	0.030417	0.024720	0.9899
RBF	Train	−4.262	10.59	0.032402	0.054177	0.989
	Test	−3.240	12.11	0.069143	0.051809	0.9449
	Total	−4.060	10.90	0.042395	0.053665	0.9808

According to the AAPREs reported in this table, the models presented in this research can be ranked in terms of accuracy as follows:

Ensemble methods>SVR>KNN>MLP-BR>MLP-LM>RBF

It is clear that the highest accuracy after Ensemble methods is related to SVR with an AAPRE of 4.95% and the highest error is related to the RBF model. Also, the KNN algorithm has relatively good accuracy and MLP-LM and MLP-BR models report close to each other and relatively acceptable accuracy.

**Figure 6:**
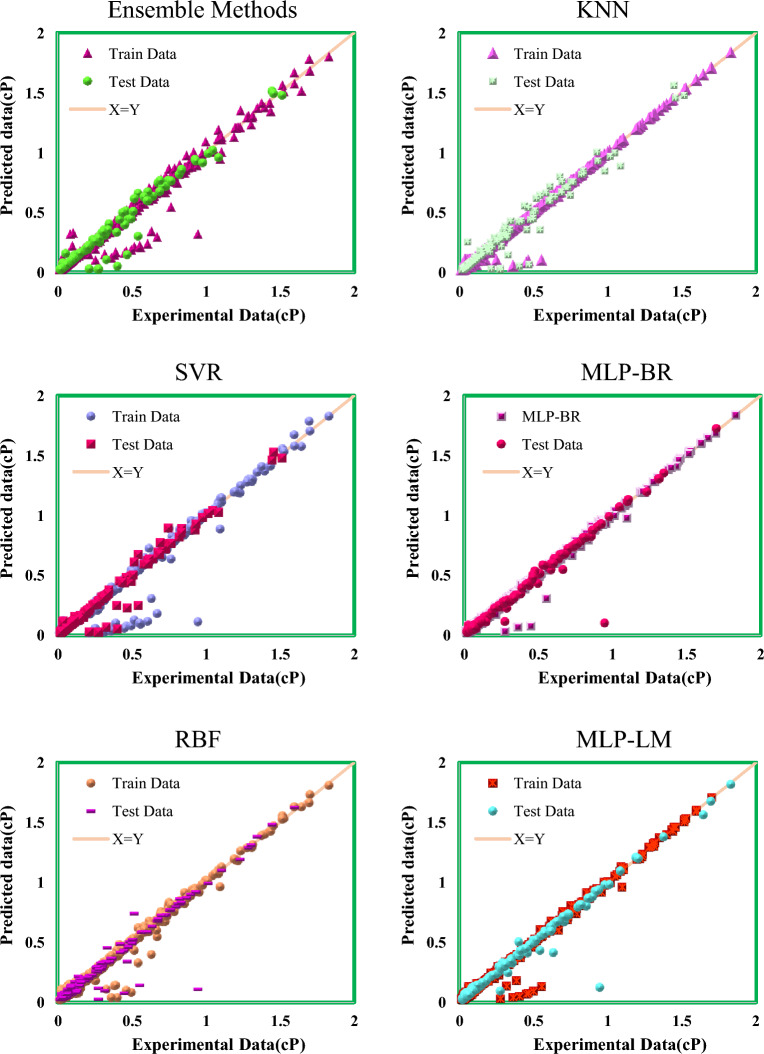
Cross-plot of presented models to predict gas-condensate viscosity.

To show the accuracy of the models graphically, the cross-plot for each model using laboratory and predicted data is presented in Fig. 6. Considering the cross-plots and high density of data around the X = Y line for all models, it can be concluded that the accuracy of the models presented in this research to predict gas-condensate viscosity is high. It is clear that the data density above and below the X = Y line is very small and it can be inferred that no underestimation or overestimation has been occurred in the models. Also in this diagram, the high compatibility of laboratory data with the data predicted by the models can be seen.

**Figure 7:**
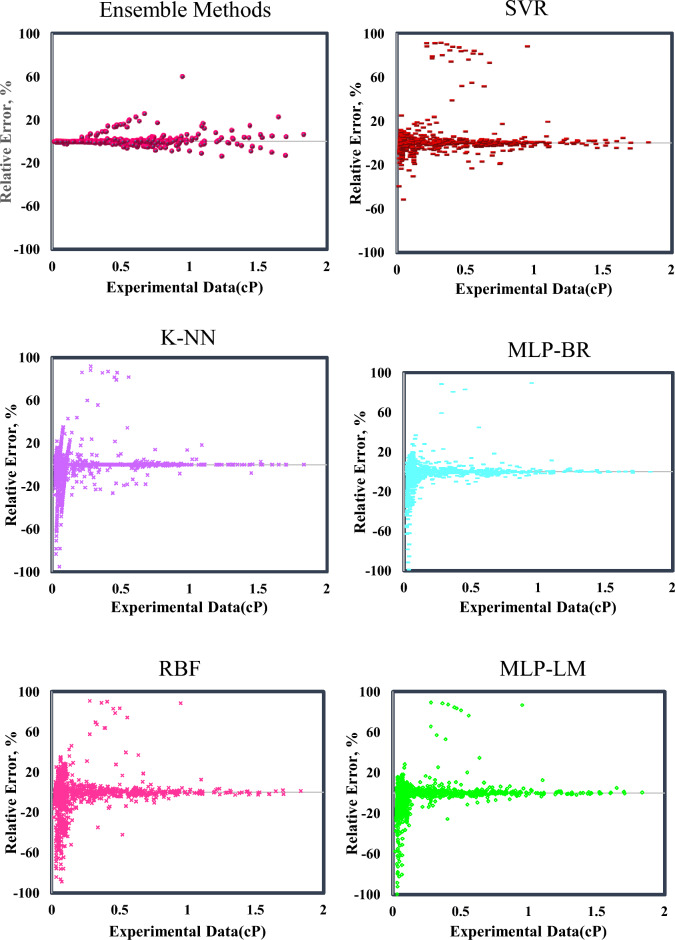
Error distribution plot of the presented models to predict gas-condensate viscosity.

The error distribution diagram based on laboratory data and the relative error of each model is plotted in Fig. 7. As can be seen, the accumulation of data around the zero-error line for Ensemble methods is more than in other models and shows low deviation and high accuracy of this model. In general, in the error distribution diagram, the higher the data scatter around the zero-error line, the lower the accuracy of the model, and the denser the data around this line, the higher the accuracy of the model. If the model has very little accuracy, the data will be completely above or below the zero-error line, indicating overestimating and underestimating, respectively.

**Figure 8:**
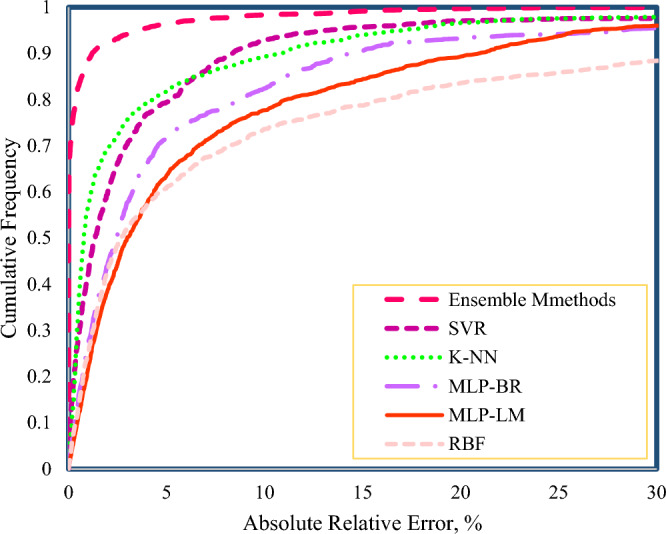
Cumulative frequency curve for the developed models in this study.

Despite the high accuracy of the models presented in this research, the introduction of the most accurate model in terms of precision is important. Figure 8 shows the cumulative diagram of the developed models, which is visually plotted for a better comparison of the models. It is observed that Ensemble methods report an error 1% for 90% of the data and have high accuracy. In addition, the accuracy of SVR and KNN models are almost equal, and for 80% of the data, they report an error of less than 5%. MLP neural networks trained with LM and BR algorithms report errors below 10% for 80% of data. Moreover, the RBF neural network reports errors below 20% for 70% of the data.

**Figure 9:**
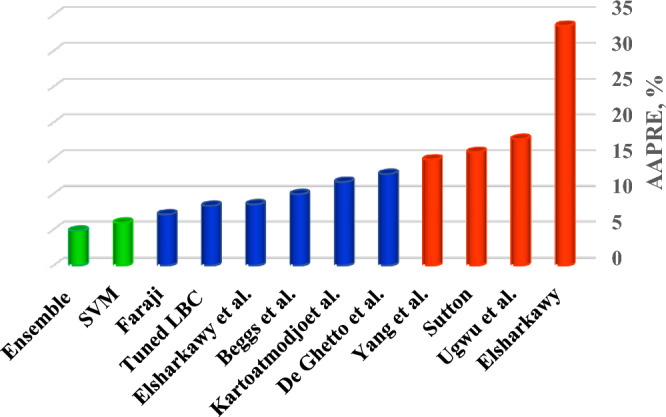
Bar chart to compare the most accurate models presented in this research and the models presented in literature.

Also, in order to check the validity of the Ensemble model as the most practical model presented, a complete comparison was done based on AAPRE with the illustrious models of literature. According to Table 4, it is clear that the most accurate model in the literature reports AAPRE of 7.23%, which is presented by Fouadi et al.^5^. Also, the Ugwu et al.^69^ models report high average absolute errors to predict viscosity. To compare these results graphically, a bar chart was presented in Fig. 9, which shows a comparison of the average absolute relative error of two of the most accurate models presented with the well-known models in the literature.

**Figure 10:**
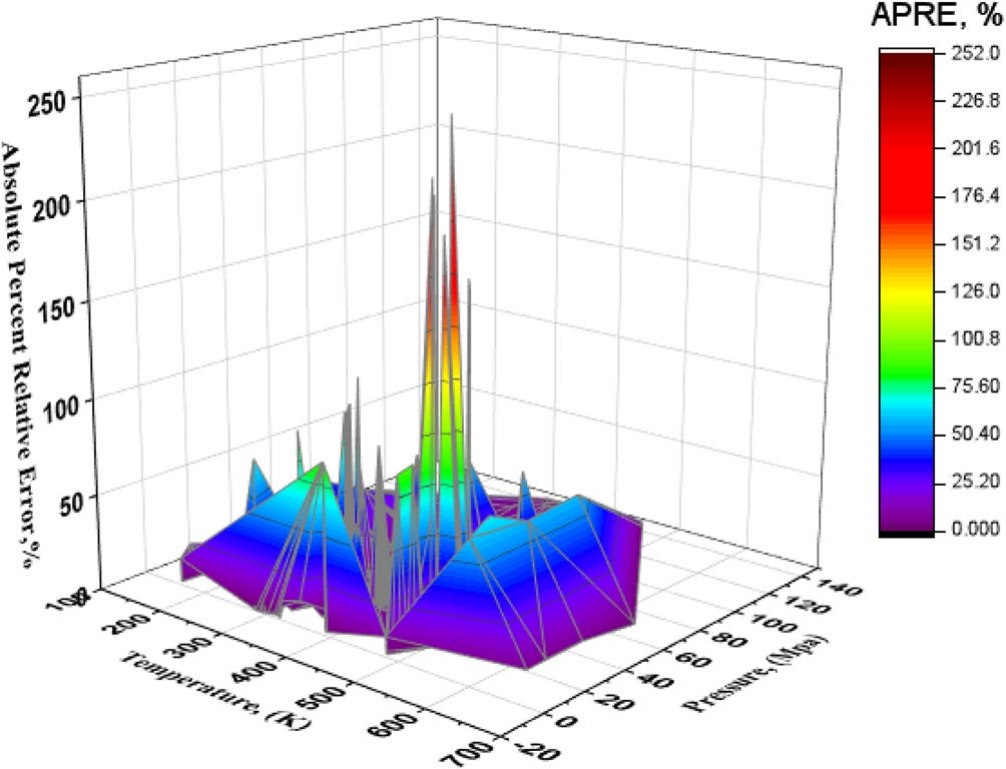
Three-dimensional diagram of AAPRE in terms of temperature and pressure for the Ensemble Methods model.

A three-dimensional graph was used to determine the points that report the most absolute error. Figure 10 shows a three-dimensional graph of the absolute error obtained by Ensemble Methods in terms of temperature and pressure. In this diagram, the peaks represent high absolute error and the smooth surfaces indicate temperature and pressure conditions that report a low absolute error. It is clear that in most temperature and pressure conditions, a low error is seen, although some points in the temperature range of 250–300 K and the pressure range of 80–100 MPa report a large absolute error of about 200%.

**Figure 11:**
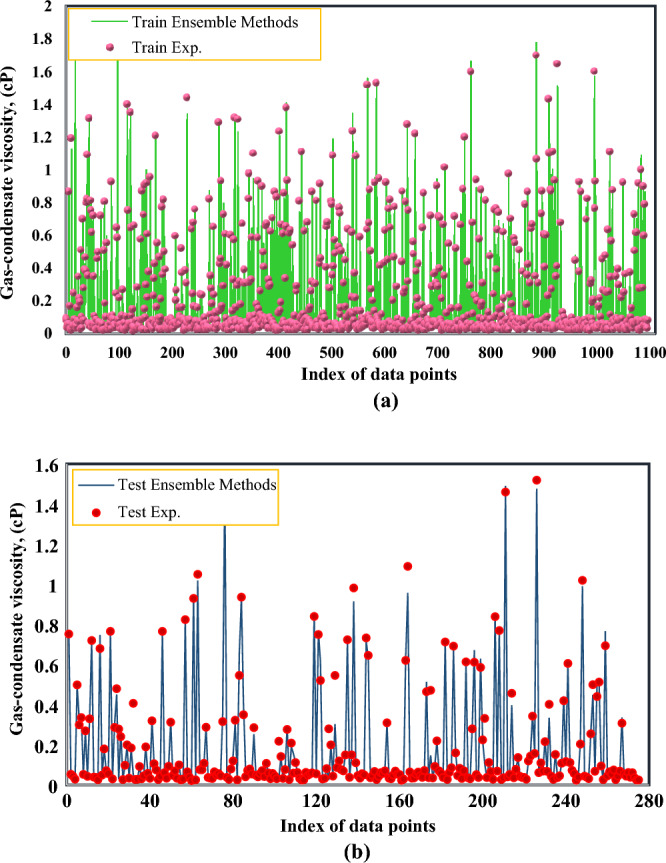
Comparison between experimental gas-condensate viscosity and predicated data using Ensemble Methods for the (**a**) Train and (**b**) Test subsets.

Figure 11 shows a good correlation between the data estimated by the ensemble methods model and the laboratory data for training and testing. This indicates a high accuracy obtained from this model.

### Sensitivity analysis

One of the most important statistical analyses is the check of the effect of input parameters on the output of the model, which is known as sensitivity analysis and uses the Pearson equation ^70,71^. The outputs of this relationship are between −1 and 1, and negative values indicate a negative effect of the parameter on the output and positive values indicate a positive effect, and the larger the value, the greater effect of the parameter on the model output, and vice versa^72^. The formula used to perform this analysis is as follows^73^:

26 $$r = \frac{{\sum_{i = 1}^{n} {\left( {I_{k,i} - \overline{I}_{K} } \right) - \left( {O_{i} - \overline{O} } \right)} }}{{\sqrt {\sum_{i = 1}^{n} {\left( {I_{k,i} - \overline{I}_{K} } \right)^{2} \sum_{i = 1}^{n} {\left( {O_{i} - \overline{O} } \right)^{2} } } } }}$$

In this regard, the number of data, *i*th input, *i*th output, mean *k*th input, and mean output are denoted by $n,\,I_{k,i} ,\,O_{i} ,\,\overline{{I_{k} }} ,\;{\text{and}}\;\overline{O}$, respectively.

**Figure 12:**
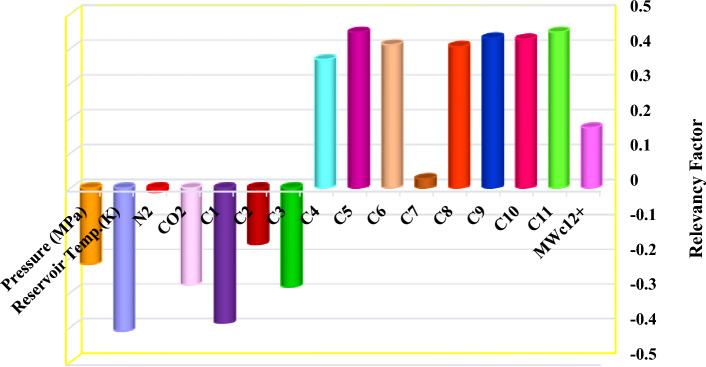
Investigation of the effect of input parameters of the most accurate model presented in this research on the viscosity of condensate.

Figure 12 illustrates the effect of model inputs on the output of Ensemble Methods. As it is clear, the most negative effect is related to the reservoir temperature and the most positive effect is related to the mole of C_11_. Also, reservoir pressure and mole of C_1_ to C_4_ as well as the mole of non-hydrocarbon components including N_2_ and CO_2_ report negative effects on viscosity, and with increasing them, the viscosity decreases. Also, the mole fraction of other condensate components from C_5_ to C_11_ and the molecular weight of C_12+_ report positive effects on the viscosity of the condensate, and with increasing them, the amount of viscosity also increases. In addition, according to the diagram, it can be seen that the mole fractions of N_2_ and C_7_ have very little effect on the viscosity of the condensate.

### Trend analysis

**Figure 13:**
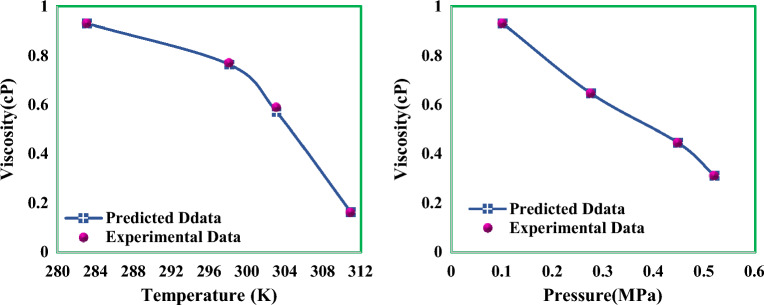
Investigation of condensate viscosity behavior against temperature and pressure changes.

The viscosity behavior of condensate at different temperatures and pressures is shown in Fig. 13. According to particle theory^74^, with increasing temperature, the distance between molecules increases which leads to a decrease in the viscosity of liquids. Changes in the viscosity of condensate with temperature can be expressed using the following formula:

27 $$\mu = ae^{ - bt}$$

In this formula, *a* and *b* are constant coefficients and a function of condensate composition. Also, according to the diagram, the condensate viscosity decreases with increasing pressure. The reason for the decrease in viscosity with increasing pressure can be related to the complex behavior of gas condensate reservoir.

### Outlier detection

There are a variety of ways to find outlier and suspected laboratory data. In this research, the Leverage technique and William diagram have been used^75,76^. Using this method, the data is placed in the valid, suspected, and outlier regions.

To draw a graph, first, the value of *H* is calculated using the following formula, and then the values of Standardized Residual (*SR*) and Hat * are calculated using the following formula^76,77^:

28 $$H = X\left( {X^{t} X} \right)^{ - 1} X^{t}$$

29 $$SR = \frac{{\left( {Output - Target} \right)}}{{\left( {\left( {1 - h} \right)^{0.5} } \right) \times RMSE}}$$

30 $$Hat^{*} = \frac{{3 \times \left( {Number\,of\,features\, + 1} \right)}}{{Number\,of\,data\,{\text{points}}}}$$

**Figure 14:**
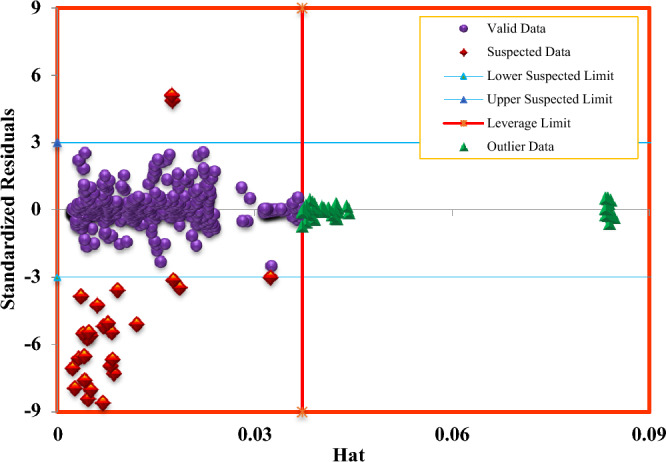
William’s plot to determine outliers and suspected data points.

Figure 14 shows the William plot obtained by Ensemble Methods. In this figure, Hat * defines the boundary between outlier data and other data, and when the value of a given data exceeds Hat *, they are out of the scope of the model. Also, data with *SR* more than 3 or less than −3 are known as suspected laboratory data and report a high error (Regardless of their hat value), and data that is in the valid area of the model, their *H*_*ii*_ is less than Hat * and their *SR* is between 3 and −3^78^. Table 5 shows outlier data indicated by the leverage technique for the Ensemble Methods. An examination of Williams plot indicates that most of the data points are located in a valid area, indicating the high validity of ensemble methods and high reliability of the data bank used in this work.

**Table 4:** Comparison of the AAPRE of the models presented in this research with the literature models.

Models	RMSE	AAPRE%
Yang et al. (2007)^3^	0.0544	14.80
Ugwu et al. (2011)^69^	0.0616	17.66
Beggs and Robinson (1975)^12^	0.0264	9.95
Kartoatmodjo and Schmidt (1994)^79^	0.0232	11.66
Elsharkawy and Alikhan (1999)^14^	0.0248	8.52
Sutton (2005)^80^	0.3673	15.84
Faraji et al. (2021)^5^	0.0194	7.12
Tuned LBC	0.0196	8.32
Ensemble methods	0.0446	4.83
SVR	0.0519	5.95

**Table 5:** Outlier data obtained by the leverage technique.

Pressure (MPa)	Reservoir Temp.(K)	N_2_	CO_2_	C_1_	C_2_	C_3_	C_4_	C_5_	C_6_	C_7_	C_8_	C_9_	C_10_	C_11_	MWC_12+_	Viscosity
0.061363	392.15	0	0	0.7355	0.08	0.0485	0.0296	0	0.0442	0.0423	0	0	0	0	271	0.066
0.040679	392.15	0	0	0.7355	0.08	0.0485	0.0296	0	0.0442	0.0423	0	0	0	0	271	0.05
0.019995	392.15	0	0	0.7355	0.08	0.0485	0.0296	0	0.0442	0.0423	0	0	0	0	271	0.031
0.073084	392.15	0	0	0.7355	0.08	0.0485	0.0296	0	0.0442	0.0423	0	0	0	0	271	0.076
0.089632	392.15	0	0	0.7355	0.08	0.0485	0.0296	0	0.0442	0.0423	0	0	0	0	271	0.087
0.082737	392.15	0	0	0.7355	0.08	0.0485	0.0296	0	0.0442	0.0423	0	0	0	0	271	0.083
3.447372	482.0051	0.0439	0.0324	0.6249	0.0421	0.0281	0.0276	0.0201	0.0185	0.024	0.0277	0.0226	0.0176	0.0125	232	0.508
10.34212	482.0051	0.0439	0.0324	0.6249	0.0421	0.0281	0.0276	0.0201	0.0185	0.024	0.0277	0.0226	0.0176	0.0125	232	0.365
45.98795	482.0051	0.0439	0.0324	0.6249	0.0421	0.0281	0.0276	0.0201	0.0185	0.024	0.0277	0.0226	0.0176	0.0125	232	0.222
48.36663	482.0051	0.0439	0.0324	0.6249	0.0421	0.0281	0.0276	0.0201	0.0185	0.024	0.0277	0.0226	0.0176	0.0125	232	0.223
42.644	482.0051	0.0439	0.0324	0.6249	0.0421	0.0281	0.0276	0.0201	0.0185	0.024	0.0277	0.0226	0.0176	0.0125	232	0.219
40.08605	482.0051	0.0439	0.0324	0.6249	0.0421	0.0281	0.0276	0.0201	0.0185	0.024	0.0277	0.0226	0.0176	0.0125	232	0.218
24.13161	482.0051	0.0439	0.0324	0.6249	0.0421	0.0281	0.0276	0.0201	0.0185	0.024	0.0277	0.0226	0.0176	0.0125	232	0.273
17.23686	482.0051	0.0439	0.0324	0.6249	0.0421	0.0281	0.0276	0.0201	0.0185	0.024	0.0277	0.0226	0.0176	0.0125	232	0.31
36.65936	482.0051	0.0439	0.0324	0.6249	0.0421	0.0281	0.0276	0.0201	0.0185	0.024	0.0277	0.0226	0.0176	0.0125	232	0.217
31.02635	482.0051	0.0439	0.0324	0.6249	0.0421	0.0281	0.0276	0.0201	0.0185	0.024	0.0277	0.0226	0.0176	0.0125	232	0.243
5.515796	390.4242	0.0708	0.0062	0.7104	0.0757	0.0348	0.0207	0.0106	0.0075	0.0107	0.0136	0.0086	0.0061	0.0041	232	0.442
48.79411	390.4242	0.0708	0.0062	0.7104	0.0757	0.0348	0.0207	0.0106	0.0075	0.0107	0.0136	0.0086	0.0061	0.0041	232	0.462
9.652643	390.4242	0.0708	0.0062	0.7104	0.0757	0.0348	0.0207	0.0106	0.0075	0.0107	0.0136	0.0086	0.0061	0.0041	232	0.384
44.54005	390.4242	0.0708	0.0062	0.7104	0.0757	0.0348	0.0207	0.0106	0.0075	0.0107	0.0136	0.0086	0.0061	0.0041	232	0.414
14.47896	390.4242	0.0708	0.0062	0.7104	0.0757	0.0348	0.0207	0.0106	0.0075	0.0107	0.0136	0.0086	0.0061	0.0041	232	0.339
41.9821	390.4242	0.0708	0.0062	0.7104	0.0757	0.0348	0.0207	0.0106	0.0075	0.0107	0.0136	0.0086	0.0061	0.0041	232	0.356
40.31357	390.4242	0.0708	0.0062	0.7104	0.0757	0.0348	0.0207	0.0106	0.0075	0.0107	0.0136	0.0086	0.0061	0.0041	232	0.312
39.43794	390.4242	0.0708	0.0062	0.7104	0.0757	0.0348	0.0207	0.0106	0.0075	0.0107	0.0136	0.0086	0.0061	0.0041	232	0.295
19.30529	390.4242	0.0708	0.0062	0.7104	0.0757	0.0348	0.0207	0.0106	0.0075	0.0107	0.0136	0.0086	0.0061	0.0041	232	0.312
24.13161	390.4242	0.0708	0.0062	0.7104	0.0757	0.0348	0.0207	0.0106	0.0075	0.0107	0.0136	0.0086	0.0061	0.0041	232	0.292
28.95793	390.4242	0.0708	0.0062	0.7104	0.0757	0.0348	0.0207	0.0106	0.0075	0.0107	0.0136	0.0086	0.0061	0.0041	232	0.277
34.47372	390.4242	0.0708	0.0062	0.7104	0.0757	0.0348	0.0207	0.0106	0.0075	0.0107	0.0136	0.0086	0.0061	0.0041	232	0.264
138.06	323.15	0	0	0.6962	0.1314	0.0919	0.031	0.0101	0.0056	0.006	0.0063	0.0042	0.0028	0.0024	191	0.119
34.56	473.15	0	0	0.42	0	0	0	0	0	0.58	0	0	0	0	0	0.092
137.94	473.15	0	0	0.6962	0.1314	0.0919	0.031	0.0101	0.0056	0.006	0.0063	0.0042	0.0028	0.0024	191	0.076
137.97	373.15	0	0	0.6962	0.1314	0.0919	0.031	0.0101	0.0056	0.006	0.0063	0.0042	0.0028	0.0024	191	0.097
41.52	473.15	0	0	0.6962	0.1314	0.0919	0.031	0.0101	0.0056	0.006	0.0063	0.0042	0.0028	0.0024	191	0.034
138	373.15	0	0	0.42	0	0	0	0	0	0.58	0	0	0	0	0	0.315
34.56	423.15	0	0	0.42	0	0	0	0	0	0.58	0	0	0	0	0	0.115
137.94	473.15	0	0	0.42	0	0	0	0	0	0.58	0	0	0	0	0	0.205
131.03	323.15	0	0	0.42	0	0	0	0	0	0.58	0	0	0	0	0	0.414
34.56	323.15	0	0	0.42	0	0	0	0	0	0.58	0	0	0	0	0	0.219
137.95	423.15	0	0	0.6962	0.1314	0.0919	0.031	0.0101	0.0056	0.006	0.0063	0.0042	0.0028	0.0024	191	0.085
137.94	423.15	0	0	0.42	0	0	0	0	0	0.58	0	0	0	0	0	0.246
34.56	373.15	0	0	0.42	0	0	0	0	0	0.58	0	0	0	0	0	0.154
41.45	323.15	0	0	0.6962	0.1314	0.0919	0.031	0.0101	0.0056	0.006	0.0063	0.0042	0.0028	0.0024	191	0.057
41.61	423.15	0	0	0.6962	0.1314	0.0919	0.031	0.0101	0.0056	0.006	0.0063	0.0042	0.0028	0.0024	191	0.038
41.72	373.15	0	0	0.6962	0.1314	0.0919	0.031	0.0101	0.0056	0.006	0.0063	0.0042	0.0028	0.0024	191	0.048
51.86	473.15	0	0	0.6962	0.1314	0.0919	0.031	0.0101	0.0056	0.006	0.0063	0.0042	0.0028	0.0024	191	0.039
121.07	323.15	0	0	0.6962	0.1314	0.0919	0.031	0.0101	0.0056	0.006	0.0063	0.0042	0.0028	0.0024	191	0.108
120.69	323.15	0	0	0.42	0	0	0	0	0	0.58	0	0	0	0	0	0.393
51.74	473.15	0	0	0.42	0	0	0	0	0	0.58	0	0	0	0	0	0.112
51.88	323.15	0	0	0.6962	0.1314	0.0919	0.031	0.0101	0.0056	0.006	0.0063	0.0042	0.0028	0.0024	191	0.065
120.7	473.15	0	0	0.6962	0.1314	0.0919	0.031	0.0101	0.0056	0.006	0.0063	0.0042	0.0028	0.0024	191	0.069
120.73	473.15	0	0	0.42	0	0	0	0	0	0.58	0	0	0	0	0	0.188
120.71	373.15	0	0	0.6962	0.1314	0.0919	0.031	0.0101	0.0056	0.006	0.0063	0.0042	0.0028	0.0024	191	0.089
51.81	323.15	0	0	0.42	0	0	0	0	0	0.58	0	0	0	0	0	0.254
51.9	423.15	0	0	0.6962	0.1314	0.0919	0.031	0.0101	0.0056	0.006	0.0063	0.0042	0.0028	0.0024	191	0.044
120.76	373.15	0	0	0.42	0	0	0	0	0	0.58	0	0	0	0	0	0.287
51.74	423.15	0	0	0.42	0	0	0	0	0	0.58	0	0	0	0	0	0.142
51.88	373.15	0	0	0.6962	0.1314	0.0919	0.031	0.0101	0.0056	0.006	0.0063	0.0042	0.0028	0.0024	191	0.052
120.68	423.15	0	0	0.6962	0.1314	0.0919	0.031	0.0101	0.0056	0.006	0.0063	0.0042	0.0028	0.0024	191	0.078
120.7	423.15	0	0	0.42	0	0	0	0	0	0.58	0	0	0	0	0	0.227
51.73	373.15	0	0	0.42	0	0	0	0	0	0.58	0	0	0	0	0	0.181
103.68	323.15	0	0	0.6962	0.1314	0.0919	0.031	0.0101	0.0056	0.006	0.0063	0.0042	0.0028	0.0024	191	0.099
69.07	473.15	0	0	0.6962	0.1314	0.0919	0.031	0.0101	0.0056	0.006	0.0063	0.0042	0.0028	0.0024	191	0.047
103.43	323.15	0	0	0.42	0	0	0	0	0	0.58	0	0	0	0	0	0.356
68.99	473.15	0	0	0.42	0	0	0	0	0	0.58	0	0	0	0	0	0.132
69.09	323.15	0	0	0.6962	0.1314	0.0919	0.031	0.0101	0.0056	0.006	0.0063	0.0042	0.0028	0.0024	191	0.076
103.51	473.15	0	0	0.6962	0.1314	0.0919	0.031	0.0101	0.0056	0.006	0.0063	0.0042	0.0028	0.0024	191	0.064

## Conclusions

In this study, an accurate model was presented to predict the viscosity of gas condensate in models presented in the literature, one of the input parameters for the development of the models is solution gas oil ratio (Rs). Measuring Rs in wellhead requires special equipment and is somewhat difficult. Also, measuring this parameter in the laboratory requires spending time and money. According to the mentioned cases, in this research, unlike the research done in the literature, Rs parameter was not used to develop the models. The input parameters for the development of the models presented in this research were temperature, pressure and condensate composition. The data used includes a wide range of temperature and pressure, and the models presented in this research are the most accurate models to date for predicting the condensate viscosity. The accuracy and validity of the models were compared with each other using statistical error parameters as well as graphically, and finally, ensemble method with an AAPRE of 4.83% was introduced as the most accurate model. Also, the accuracy of the best models presented in this study was compared with well-known models of literature. It was observed that some models in the literature report good accuracy only in limited conditions of temperature and pressure and have a high error at different conditions of temperature and pressure. Sensitivity analysis showed that the most negative effect of inputs on the viscosity of condensate is related to the reservoir temperature and the most positive effect is related to the mole fraction of C_11_. Also, reservoir pressure and mole fraction of hydrocarbon components from C_1_ to C_4_ as well as the weight fractions of non-hydrocarbon components including N_2_ and CO_2_ report negative effects on viscosity and with increasing them, the viscosity decreases. Also, the mole fraction of other condensate components from C_5_ to C_11_ and the molecular weight of C_12+_ report positive effects on the viscosity of the condensate, and with increasing them, the amount of viscosity also increases. Finally, the great reliability of the employed data set for modeling and excellent validity of ensemble methods were proved by applying the Leverage approach, and suspected data were reported in a table.

## Data Availability

The databank utilized during this research is available from the corresponding author on reasonable request.
